# Shared decision making for psychiatric medication management: beyond the micro‐social

**DOI:** 10.1111/hex.12392

**Published:** 2015-08-10

**Authors:** Nicola Morant, Emma Kaminskiy, Shulamit Ramon

**Affiliations:** ^1^ Division of PsychiatryUCLLondon; ^2^ Department of PsychologyAnglia Ruskin UniversityCambridgeUK; ^3^ Department of Education and Social CareAnglia Ruskin UniversityCambridgeUK

**Keywords:** doctor–patient communication, mental health, patient involvement, psychiatric medication, psychiatry, shared decision making

## Abstract

**Background:**

Mental health care has lagged behind other health‐care domains in developing and applying shared decision making (SDM) for treatment decisions. This is despite compatibilities with ideals of modern mental health care such as self‐management and recovery‐oriented practice, and growing policy‐level interest. Psychiatric medication is a mainstay of mental health treatment, but there are known problems with prescribing practices, and service users report feeling uninvolved in medication decisions and concerned about adverse effects. SDM has potential to produce better tailoring of psychiatric medication to individuals' needs.

**Objectives:**

This conceptual review argues that several aspects of mental health care that differ from other health‐care contexts (e.g. forms of coercion, questions about service users' insight and disempowerment) may impact on processes and possibilities for SDM. It is therefore problematic to uncritically import models of SDM developed in other health‐care contexts. We argue that decision making for psychiatric medication is better understood in a broader way that moves beyond the micro‐social focus of a medical consultation. Contextualizing specific medication‐related consultations within longer term relationships, and broader service systems enables recognition of the multiple processes, actors and agendas that shape how psychiatric medication is prescribed, managed and used, and which may facilitate or impede SDM.

**Conclusion:**

A broad conceptualization of decision making for psychiatric medication that moves beyond the micro‐social can account for why SDM in this domain remains a rarity. It has both conceptual and practical utility for evaluating research evidence, identifying future research priorities and highlighting fruitful ways of developing and implementing SDM in mental health care.

## Introduction

Shared decision making (SDM) about treatment options is now a widely recognized aspect of patient‐centred care that has become a modern health‐care ideal internationally.^1^ In SDM patient and clinician discuss treatment options in a two‐way exchange of information and knowledge (formal and experiential), and together decide on a course of action.^2^ This collaborative process is based on mutual respect, open communication and consideration of individual preferences and values. In the UK, SDM is promoted in government policies,^3^ good practice guidance^4^ and initiatives to shape standard clinical practice.^5^ A large body of research has shown positive effects on patient satisfaction, treatment adherence, health status and health inequalities.^6^, ^7^, ^8^

In the field of mental health,^1^ SDM has received much less attention^7^, ^8^, ^9^ and remains a relative rarity in standard clinical practice.^10^ A systematic review of SDM interventions in mental health found only two eligible studies and concluded that further research was urgently needed.^11^ However, there is growing interest in SDM in mental health, which has increasingly featured in mental health policy and good practice rhetoric,^12^, ^13^ and fits well with the ‘recovery’ approach that characterizes modern mental health‐care ideals in many developed countries.^14^, ^15^, ^16^, ^17^ This patient‐centred orientation promotes self‐management and aims to support people to live well with and beyond their mental health problems, combining formal treatments with other well‐being strategies.^18^ Experiential knowledge is valued, and more equal, collaborative practitioner–user relationships are promoted.

Despite these recent developments, shadows of a darker past still characterize many aspects of standard mental health practice. Forms of coercion from subtle persuasion to compulsory hospitalization or community treatment orders (CTOs) are still relatively common. Many mental health service users^2^ remain disempowered, feel they have little voice in treatment decisions, or that these are not made in their interests, and experience stigma.^19^ Whilst there may be moments of genuine lack of capacity, meaningful dialogue can also sometimes be compromised by practitioners' assumptions about lack of insight associated with mental health problems. This may exaggerate inequalities between service users' experiential knowledge and the scientific knowledge base of practitioners. These dynamics are most common when mental health problems are acute or severe (although they may not be explicitly acknowledged by service providers), but discrete experiences of threatened, perceived or actual coercion can erode service users' long‐term ability to trust and engage positively with services.

Our focus in this paper is specifically on SDM for psychiatric medication management within specialist mental health services. Psychiatric medication (antipsychotics, mood stabilizers, antidepressants and anxiolytics) is a mainstay of treatment for mental health problems, particularly for psychotic disorders and acute mental health crises. Again, there is a disjuncture between policy ideals and much of standard clinical practice. Whilst the value of patient choice and active involvement in medication decisions is emphasized in practice recommendations and policies,^13^, ^20^, ^21^, ^22^, ^23^ mental health service users commonly report feeling uninvolved in decisions about medication and often feel they lack choice.^10^, ^13^, ^24^, ^25^ Medication can be bound up with forms of coercion: Service providers may persuade or pressure users to take medication, or to have long‐acting ‘depot’ injections if they do not take oral medication as practitioners would like. Taking medication as prescribed can be a requirement of legally binding CTOs, or a determining factor of voluntary or compulsory hospital admission status.

Medications such as antipsychotics, mood stabilizers and antidepressants are powerful drugs that are usually taken for long periods of time (often decades) and can produce a wide range of wanted and unwanted physical and psychological effects. Prescribing the most appropriate type and dose of medication is a complex process of negotiating uncertainties in diagnosis, individual responses and patient acceptability. These challenges make the combination of users' experiential knowledge and practitioners' clinical knowledge within SDM a valuable approach in achieving optimal medication use for an individual. Simultaneously, the potential for psychiatric medication to be linked with coercion imposes different meanings and implications on these discussion compared to other forms of medicine‐taking.

In this paper, we suggest that several characteristics of mental health care (and the use of psychiatric medication within this) mean it is problematic to uncritically apply conceptualizations of SDM developed in other domains of health care to mental health contexts. This is because features such as disempowerment, forms of coercion, questions about service users' ‘insight’ and stigma, that are more prominent than in other health‐care contexts, impact on the processes and possibilities of SDM. The majority of SDM work has had a primarily micro‐social focus on doctor–patient consultations.^6^, ^26^, ^27^, ^28^ We propose a conceptualization of decision making for psychiatric medication that moves beyond the micro‐social, and contextualizes doctor–patient interactions within longer term relationship and treatment processes, and broader organizational contexts in which many of the unique aspects of mental health care are lived out. This area is in its infancy, and the research base is small^11^ with considerable methodologically and disciplinary diversity, and little consensus on objectives, target groups or outcomes. Therefore, a conceptual review that promotes critical thinking and conceptual clarification is timely, and arguably has more utility at this stage than a conceptually uncritical systematic review.

After a brief review of the prescription and management of psychiatric medication the components of our broader conceptualization are set out. We use this to integrate and critically evaluate existing evidence on SDM for psychiatric medication and to identify directions for future research and clinical practice.

## Psychiatric medication: prescription and use

### Experiences of taking psychiatric medication

Although many service users report benefits of psychiatric medication, concerns about the impact of adverse effects^3^ on life quality, well‐being and social functioning are common.^29^, ^30^ Common negative effects include weight gain, drowsiness and mental clouding, reduced libido, involuntary movements and diabetes. Users can often find themselves swapping symptoms of mental ill health for another set of problems.^24^ Consequently, not taking medication as prescribed is widespread: between a third and a half of people do not take prescribed psychiatric medication at all, take less than the prescribed dose or stop taking it abruptly.^31^ These practices, especially abrupt stopping of medication, can be associated with deteriorations in mental health and increased likelihood of relapse.^32^

Psychiatrists are therefore justifiably wary of users not taking medication as prescribed, but they often fail to recognize this as part of positive self‐management strategies. Over time, many people learn to successfully tailor their medication in response to mental states and life events, integrating this into broader recovery and ‘personal medicine’ strategies.^33^, ^34^ ‘Purposeful non‐adherence’ is a common strategy to minimize medication intake that is seldom disclosed to prescribers.^35^, ^36^ Psychiatric medication carries complex and ambivalent meanings linked to identity and sense of self.^37^ Not taking medication may be an attempt to regain control in response to negative or coercive experiences of mental health care: it can be a service user's ‘trump card’, their only and ultimate source of resistance in a context of experienced powerlessness and lack of choice.

### Prescribing practices

Several concerns have been raised recently about high dose and overprescribing of psychiatric medication, and failure to follow prescribing guidelines. A significant proportion of UK users of antipsychotics are prescribed more than 100% of the recommended maximum dose.^10^, ^20^ Doses are often increased during a mental health crisis, but not reduced once the crisis is resolved. Polypharmacy is common,^10^ bringing greater adverse effects risks associated with drug interactions or higher overall doses.^38^

Doubts about the balance of efficacy vs. adverse effects have been expressed, with arguments that the efficacy of antipsychotics and other medications may have been overestimated, and the seriousness of adverse effects underestimated.^39^ Adverse effects are likely to be more severe on high doses, and some can be irreversible and associated with serious long‐term negative health consequences.^40^, ^41^ The dominant disease‐targeting model of psychiatric medication has been questioned, giving greater weight to users' subjective experiences as the target of treatment, not just an interesting by‐product^42^. Research shows that many people can live well with no or low doses of medication, often by developing positive strategies for managing symptoms or within strong supportive networks.^43^, ^44^ Within the UK public mental health system, there is little development of such approaches.

### Prescriber–user discussions about medication

Medication is one of the most important decisional domains for mental health service users,^45^ but they report receiving little or insufficient information about adverse effects,^10^, ^29^ difficulties in raising medication concerns with psychiatrists and low levels of involvement in medication decisions.^10^, ^13^, ^25^, ^36^ Micro‐analytic studies of psychiatric consultations support this. In several domains including medication, psychiatrists rarely use communication strategies that encourage SDM (although wide variations are found) and often use strategies to resist engagement with users' concerns and questions.^46^, ^47^ When discussing antipsychotics, they frequently fail to address users' concerns about sedation and mental clouding, sometimes by questioning the validity of patients' interpretations.^48^ The ‘option set’ (the choices from which to decide) is often unilaterally defined by psychiatrists who may steer users towards a particular decision or mark one course of action as best.^49^ These studies suggest that psychiatric consultations often fail to support patient choice and SDM for psychiatric medication and can be unequal in terms of participants' access to information and means of persuasion. To understand this, we need to consider the multilevel factors that contribute to these processes.

## A conceptual model of decision making for psychiatric medication

**Figure 1:**
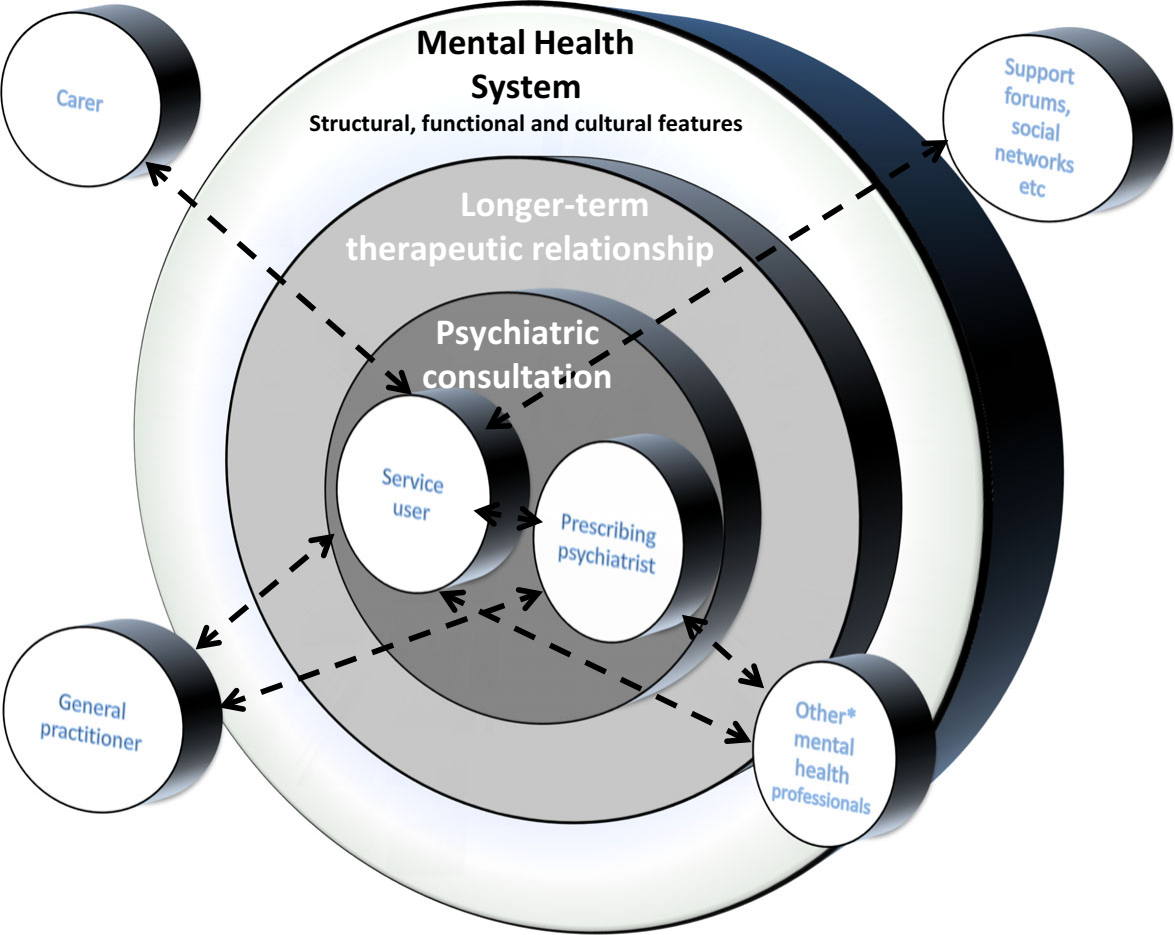
Decision making for psychiatric medication. *e.g. community psychiatric nurse, social worker, pharmacist, psychologist, peer support worker.

Our conceptualization of decision making for psychiatric medication builds on and extends the arguments of other commentators for broader SDM models that move beyond the micro‐social, and includes factors such as professional ethics, accountability and treatment option constraints.^26^, ^27^, ^50^ It provides a structural representation of the domains within which features of mental health care that are unique, or more exaggerated than in other health‐care domains, operate (e.g. forms of coercion, questions about ‘insight’, user disempowerment) and impact on decision‐making processes and possibilities for SDM (Fig. 1). The micro‐social processes of a psychiatric consultation are embedded within a longer term relationship, and a service context that includes other key players (professional and non‐professional), and functional and cultural features of the mental health‐care system. This is dynamic over time (so the three‐dimensional components in Fig. 1), in recognition that mental ill health and its management may evolve through periods of wellness and relapse, and SDM within this is a long‐term process.

We propose this conceptualization as a heuristic framework that informs our research on SDM for psychiatric medication^51^, ^52^ and may enable other researchers to clarify and critically consider relevant interactive, relational and systemic processes identified by work in shared decision making, mental health and medical sociology. It should not, however, be considered as a tool to guide specific clinical encounters. With modifications, it may also help researchers to conceptualize other treatment decisions in mental health or decision making for psychiatric medication managed in primary care settings. In the following sections, we discuss the inter‐related components of this model.

### The psychiatric consultation

At the micro‐social level, we highlight two characteristics of medication discussions between practitioners and service users that may differ from standard models of SDM developed in other areas of health care. First, the status and value of mental health service users' experiential knowledge is ambivalent. On the one hand, users are increasingly recognized as ‘experts by experience’ within recovery‐oriented practice, and their accounts of subjective experiences are acknowledged as essential to judging the impact of medication. One the other hand, if judged to lack capacity or insight, the validity of their views and subjective experiences can be questioned or devalued. This may lead to treatment preferences being discounted, over‐ruled or, at its extreme, choice being removed. This seldom happens in other domains of health care, in which patients' views are generally considered valid even if they disagree with practitioners.

Second, standard SDM models, in which building and reaching consensus about treatment are defining characteristics,^2^ fail to capture the complex and conflictual processes that characterize some psychiatric consultations. When practitioner and service user fundamentally disagree about whether the person is mentally ill or medication is desirable, a shared decision acceptable to both parties may not be achievable. Treatment decisions that are weighted towards risk or safety concerns, and based on more than simply the interests of the individual, may place limitations on SDM, although services may be reluctant to acknowledge this explicitly. For example, choices between types of medication may be retained, but decisions to not take medication, or to receive medication in tablet rather than depot form may be removed from the option set offered by practitioners. Attempting to maintain partnerships despite disagreements, and encouraging respectful and open discussions can allow these more challenging situations to conform to *processes* of SDM that may confer benefits,^53^ even if a shared decision as an *outcome* is not possible, such as when compulsory treatment is enforced. Thus, the possibilities for SDM within a single psychiatric consultation relate to agendas in the broader organizational and social context of mental health care.

### Decision making within therapeutic relationships

Strong therapeutic relationships between mental health service users and practitioners are central to users' experiences and treatment outcomes.^54^ Similarly, SDM processes rely on good therapeutic relationships between practitioner and service user,^16^, ^55^ allowing discussions to broaden from simple ‘technical’ discussions of pros and cons, to co‐constructing understandings of medication in relation to a person's life circumstances and goals.^27^ In turn, this may contribute to further enhancing partnerships and collaborations over the longer term. Within a recovery‐oriented framework, giving greater weight to service users' experiential knowledge shifts the practitioner's role from authority to coach offering specialist knowledge,^15^ such that decisions about medication become ‘an open experiment between two co‐investigators’.^16^ As people's understanding of their mental ill health and its management develops over time, they may become increasingly empowered to participate as equal partners in discussions and choices about medication. Thus, SDM has the potential to be not just a means of deciding on treatment, but an important part of treatment itself, promoting agency and self‐management, and potentially contributing to raising trust and improving therapeutic relationships. Progressive development of SDM within positive therapeutic relationships may protect against experiences of disempowerment or alienation from services in crisis situations, or when a person's ability to participate in decisions is compromised.

Conversely, the association between therapeutic relationships and medication management practices can sometimes have mutually reinforcing negative impacts. Detrimental effects on therapeutic relationships have been found following 2 years of taking medication by long‐acting injection,^55^ and poor relationships with a prescriber and experiences of coercion during admission predict negative attitudes to antipsychotic treatment.^56^ Such negative experiences erode trust and may undermine future possibilities for SDM. Our research shows that fear of coercion is a barrier to mental health service users' involvement in medication decisions, and prevents disclosure of symptoms or personal adaptations to medication use.^51^

### Involvement of multiple stakeholders

Whilst the psychiatric consultation may be where final decisions about medication are made, much of the emotional, informational and evaluative work behind treatment preferences occurs outside this context, and is typically ‘distributed’ within social networks.^53^, ^57^ Family members can often collaborate positively in these processes, although their role has been under‐acknowledged in both SDM research and mental health care. As well as local support networks, internet forums are increasingly sources of emotional support, knowledge gain and confidence‐building.

The role of other health practitioners has also been under‐acknowledged in SDM models.^58^ Psychiatric nurses, social workers, psychologists and peer support workers may meet mental health service users more regularly than prescribing psychiatrists, providing opportunities to discuss medication.^59^, ^60^ Non‐medical practitioners can make various contributions to medication decisions, including exploring concerns, preferences, aspirations and perceived benefits and problems of medication; providing support to seek out or understand medication information; helping service users prepare for psychiatric consultations by clarifying what they want to discuss; or accompanying them to consultations. In these ways, they may amplify the voice of service users who lack confidence to express their views honestly with psychiatrists. Our work suggests psychiatric nurses and care co‐ordinators see themselves as ‘walking a shared journey’ with service users, are positive about SDM and may recognize the value of service users' experiential knowledge more than psychiatrists,^51^ but often feel under‐confident about having sufficient or appropriate medication knowledge to discuss choices in depth.^52^ Primary care physicians or general practitioners (G.Ps) may also be involved in monitoring or prescribing medication, and, for the antidepressants and anxiolytics, are often solely responsible for these tasks. However, they may be less knowledgeable about psychiatric medication than their psychiatric colleagues, and reluctant to reduce or change doses recommended by psychiatrists.

The roles of various practitioners and supporters may vary across a person's illness trajectory as they move between different part of the health system (for example, between primary and specialist services, or between inpatient and community‐based care). Across service settings, users may encounter different opinions about medication and involvement in decision making, shaping their expectations for each new clinical encounter. Medication‐related decision making typically involves numerous knowledge‐based, values‐based and interactive processes distributed over a network of stakeholders and supporters across contexts and time, with the service user as the constant factor.

### The mental health system as the context for SDM

We have already discussed features of contemporary mental health‐care systems that may facilitate SDM (policy rhetoric in support of patient choice, and recovery‐oriented approaches), and those unique to mental health care that present challenges to SDM (forms of coercion, questions about insight and capacity). Processes and possibilities of SDM specifically for psychiatric medication may be shaped by other systemic factors including: a short‐term and risk‐averse service culture that prioritizes relapse avoidance over the potential harm of long‐term medication use; reliance on biomedical models of mental illness that prioritize medication and medical expertise over other treatment strategies; dominance of a disease‐targeting model of psychiatric medication that may obscure alternative explanations^42^; professional pessimism about long‐term prognosis; lack of prescriber confidence about reducing or stopping medication^61^; the relationships of psychiatry with the pharmaceutical industry; psychiatry's broader societal role in regulating behaviour, and the use of medication in this; and (particularly in the current UK context) resource limitations that reduce regular contact with psychiatrists.

## Locating existing evidence within this conceptualization

This conceptualization of decision making for psychiatric medication can be used to evaluate, position and integrate relevant research from a range of disciplinary areas.

### Stakeholders' preferences and concerns

In keeping with the micro‐social focus of much SDM research,^26^ a considerable amount of research has explored practitioners' and service users' preferences and concerns regarding medication‐related decision making. These are key facilitators or barriers to implementing SDM. Many mental health service users want more involvement in treatment decisions, and medication decisions in particular.^24^, ^25^, ^62^ Whilst there are individual differences in relationships with medication^35^ and decision making preferences,^63^ involvement preferences are not static traits, but related to experience and stages of illness.^64^, ^65^ Service users often prefer a more directive practitioner style in times of crisis.^51^ They may become more confident users of both medication and services over time,^37^ especially if supported to develop greater autonomy and self‐management skills.^15^ This supports our dynamic conceptualization and suggests that SDM is not a ‘one size fits all’ process but should be tailored to the preferences, needs and illness stage of individuals.

For their part, psychiatrists express ‘cautious willingness’ about SDM,^9^ and some report already practicing aspects of SDM.^61^, ^66^ Practitioners' reservations are most commonly about service users' competence to participate in decision making at some stages of their illness,^61^, ^67^ and that SDM will require more time.^58^, ^60^, ^68^ Some psychiatrists think medication decisions are less suitable for SDM than other care decisions,^66^ and fear that discussing adverse effects could discourage medication use.^61^ Little is known about the views of non‐medical mental health practitioners,^68^ or about family carers' views.

### Interventions to enhance SDM

Compared to the wealth of SDM work in other health domains,^6^ only a small number of studies exist in mental health.^11^ Those focusing on or including medication decisions have produced some positive results using interventions targeted at various practitioners (not just prescribing psychiatrists). A randomized trial of SDM training for nurses and psychiatrists in inpatient settings showed that nursing support to use a decision aid in advance of psychiatric consultations increased acutely unwell service users' knowledge and decisional involvement.^69^ However, involvement was not sustained over time, a fact attributed by the authors to the one‐off nature of the intervention. Other studies have recognized the value of interventions targeting longer term processes. For example, structuring meetings around users' needs and concerns in several domains including medication over a 12‐month period produced positive effects on subjective life quality, unmet needs and treatment satisfaction.^70^ Training care co‐ordinators in effective medication management using SDM principles led to improvements in clinical symptoms and service user involvement, and reductions in antipsychotic doses, depots and polypharmacy after 9 months.^71^ However, a sole focus on practitioner training and reliance on practitioners to encourage user participation risk the impact on service users being potentially diluted by poor practitioner implementation and omit the training needs of service users to enable confident and active decisional involvement.

A promising intervention that targets service users directly is ‘Common Ground’.^72^ Developed in the USA, this provides computerized recovery‐oriented information and medication‐related decision aids. A report on the person's concerns, preferences and goals is reviewed in a psychiatric consultation and used to guide subsequent courses of action. Increased involvement in medication decisions and disclosure of information and concerns that users found difficult to tell psychiatrists directly were found.^72^ When implemented in 12 outpatient clinics, the programme was used by 85% of service users and was associated with increases in self‐reported overall health and perceived helpfulness of psychiatric medication, and reductions in symptoms and concerns about negative medication effects.^73^ This suggests a valuable role in improving the tailoring of medication to individuals' needs.

Our conceptualization of decision making for psychiatric medication suggests that interventions *directly* targeting both sides of practitioner‐service user dyads (or indeed all stakeholders in decisional processes) have the greatest potential to impact on established roles and interactive processes in a psychiatric consultation. Our current ‘ShiMME’ project is unique in providing SDM training simultaneously to service users, psychiatrists and multidisciplinary care co‐ordinators.^52^ Both ‘ShiMME’ and ‘Common Ground’ avoid the pitfalls of an exclusively micro‐social focus by taking account of facilitators and barriers to SDM within the mental health system. Whilst inequalities of knowledge and power can be barriers to involvement in general health care,^74^ our conceptualization highlights how greater levels of disempowerment, stigma and coercion in mental health settings may exaggerate barriers to involvement. Therefore, peer support and confidence‐building are central to ‘ShiMME’ training which is provided in group format by user‐trainers.^52^ Institutional inequalities are also addressed by including user perspectives in practitioner training. In ‘Common Ground’ peer workers provide support in using the computer package and exploring and articulating concerns. Both projects capitalize on facilitative factors within the organizational culture, by integrating SDM with other well‐being, recovery and self‐management strategies. ‘Common Ground’ also engages with structural limitations of the organizational context by reconfiguring outpatient clinics to include a ‘Decision Support Centre’ and scheduling time in advance of psychiatric appointments for service users to work with peers within this. This enables consultation times to remain the same whilst focussing them more efficiently on service users' concerns.

## Implications for research and clinical practice

More research is needed on interventions to promote SDM for psychiatric medication, and on implementation and sustainability issues. Specific gaps in our knowledge include the potential contributions of non‐prescribing mental health practitioners, G.Ps, peer workers and family carers, and the feasibility and limitations of SDM in acute care settings and at times of mental health crisis when coercion is most likely.

Based on our broad conceptualization, SDM interventions that target all involved parties (not just one member of practitioner–service user dyads) and decision making over time (rather than single or one‐off decisions), and acknowledge structural, cultural and functional facilitators and barriers in the mental health system are most likely to produce positive effects. For example, simply providing trustworthy and understandable medication information or decision aids may be insufficient to enable active and equal service user participation in decisions, unless accompanied by strategies to counter existing asymmetries with practitioners.^74^ Confidence‐building and empowerment should be core components of SDM initiatives for service users. SDM initiatives also need to be compatible with current mental health service configurations. For example, in the UK context, typically infrequent contact with a psychiatrist may offer limited scope for all aspects of SDM, but opportunities exist for other practitioners to implement components of SDM, such as exploring values and goals or accessing user‐friendly information.

The training needs of these practitioners in medication knowledge and SDM‐related skills need to be recognized and addressed. This should include learning more about service users' experiences of medication, the positive strategies they use to tailor medication intake to individual needs and life circumstances, their use of other well‐being strategies and their social support and informational resources. Training should explicitly address how the standard ‘script’ of practitioner–service user meetings is challenged by SDM, and the dilemmas raised by marrying up SDM with professional accountability and risk considerations in complex or conflictual clinical situations.^22^, ^40^ Although many practitioners believe they already practice elements of SDM,^58^ discrepancies with service users' ratings of involvement suggest they may be unaware of institutional or individual failures to support involvement. Existing provider–user inequalities in mental health, and ways of valuing both scientific and experiential forms of knowledge should be considered. Given that practitioners and users often disagree about the role and value of psychiatric medication, training should consider how interactive processes that are part of SDM (e.g. exploring values, valuing experience) can be maintained when agreement cannot be reached, or when interests other than the service user's shape treatment decisions. This is important because the strongest desire for more involvement has been found among those with negative views of psychiatric medication and treatment,^62^ who potentially have much to gain from new forms of dialogue or engagement with service providers. Finally, professionals should learn to encourage and offer SDM as much as possible, whilst being sensitive to individual preferences and their variations related to current illness status.^75^

Collectively, service providers should consider: how decision aids and other SDM tools (e.g. medication diaries, comparative medication information) can be best integrated into clinical practice; resources to help service users prepare for time‐limited and infrequent consultations with prescribers; and whether service reconfigurations may be necessary to support SDM. Acceptability of SDM initiatives and implementation in clinical practice may be greater if practitioners' preconceptions about SDM are acknowledged. Resistance may stem from fears of relapse if users stop taking medication; the balance between positive risk‐taking and professional accountability; and ceding professional power. Overstretched practitioners' concerns about SDM requiring additional time may be allayed by evidence that this is not the case,^47^, ^58^, ^69^, ^70^ especially if accompanied by service reconfigurations.^72^

## Conclusions

SDM has the potential to alleviate problematic aspects of current psychiatric medication management. It offers greater choice and consideration of a broader range of treatment options and may produce better tailoring of medication to individuals' needs, preferences, lifestyle and stage of illness, with knock‐on effects on health and social functioning. Medical support of graded reductions or changes in medication may be more successful and less likely to lead to relapse than if users unilaterally decide to stop taking medication. Fundamentally, people are more likely to stick with a course of action they are happy with, or feel they have been involved in deciding upon.

Despite this, SDM for psychiatric medication remains an exception rather than the norm in clinical practice. Our conceptual model of decision making for psychiatric medication has potential to explain this. We have shown that providing a more sophisticated account of the multilevel factors shaping medication decisions than existing SDM models that have a de‐contextualized focus on micro‐social processes enables us to:

1. highlight features of the mental health system and psychiatric medication management that differ from, or are more exaggerated than in other health care domains (e.g. the potential for coercion, the status of experiential knowledge), and the impact of these on decision‐making processes;
2. incorporate both the current realities of psychiatric medication management and more collaborative forms of these processes;
3. highlight multilevel facilitators and barriers to SDM, and changes in processes and practices at interactive, relational and systemic levels needed to develop more shared forms of medication management;
4. integrate a broad range of theoretical and empirical work relevant to this topic from mental health research, medicine and medical sociology.

By adopting a broader conceptual framework, we can view SDM for psychiatric medication as entailing a number of related processes both within, and also *beyond* the psychiatric consultation: service users being provided with, or autonomously seeking out medication information, or being supported to do so by individuals and social networks within and beyond the mental health system; acquiring confidence to voice their medication experiences and preferences, and potentially disagree with prescribers; and collaborative co‐investigations of medication options between a service user and one or more practitioners. Many of these process challenge established provider–user roles and relationships and may require organizational and cultural shifts. Our model aims to facilitate conceptual and practical developments, and may help to narrow the current gap between theoretical and policy ideals, and clinical realities in an important area of mental health practice.

## Notes

1 We focus in this paper on specialist mental health services, whilst acknowledging that many common mental health problems such as depression and anxiety are managed exclusively in primary care. Some, but not all, of our arguments may apply to primary care settings.

2 We prefer ‘service user’ over ‘patient’ as this is the most commonly used term in mental health, and confers a more active role on the person.

3 We prefer the terms ‘adverse’ or ‘negative’ effects over the more commonly used ‘side effects’, as this risks diminishing their significance and centrality in users' lives.
